# TLK1>Nek1 Axis Promotes Nuclear Retention and Activation of YAP with Implications for Castration-Resistant Prostate Cancer

**DOI:** 10.3390/cancers16162918

**Published:** 2024-08-22

**Authors:** Damilola Olatunde, Arrigo De Benedetti

**Affiliations:** Department of Biochemistry and Molecular Biology, The Feist Weiller Cancer Center, Louisiana State University Health Shreveport, Shreveport, LA 71103, USA; damilola.olatunde@lsuhs.edu

**Keywords:** YAP regulation, TLK1, NEK1, CRPC progression, PCA models, tumors

## Abstract

**Simple Summary:**

Treatment with ARSI results in an increased expression of the TLK1B splice variant and consequent activation of the TLK1>NEK1>YAP axis. We now provide important details of the significance of the NEK1-mediated phosphorylation of the YAP-Y407F residue for its nuclear localization and transcriptional output. Furthermore, we study the effect of the pharmacologic inhibition of this pathway in a xenograft model that readily converts from androgen-dependent to castration-resistant (CRPC) prostate cancer, as a first approach to translating our work toward a clinical investigation.

**Abstract:**

Despite some advances in controlling the progression of prostate cancer (PCa) that is refractory to the use of ADT/ARSI, most patients eventually succumb to the disease, and there is a pressing need to understand the mechanisms that lead to the development of CRPC. A common mechanism is the ability to integrate AR signals from vanishing levels of testosterone, with the frequent participation of YAP as a co-activator, and pointing to the deregulation of the Hippo pathway as a major determinant. We have recently shown that YAP is post-transcriptionally activated via the TLK1>NEK1 axis by stabilizing phosphorylation at Y407. We are now solidifying this work by showing the following: (1) The phosphorylation of Y407 is critical for YAP retention/partition in the nuclei, and J54 (TLK1i) reverses this along with YAP-Y407 dephosphorylation. (2) The enhanced degradation of (cytoplasmic) YAP is increased by J54 counteracting its Enzalutamide-induced accumulation. (3) The basis for all these effects, including YAP nuclear retention, can be explained by the stronger association of pYAP-Y407 with its transcriptional co-activators, AR and TEAD1. (4) We demonstrate that ChIP for GFP-YAP-wt, but hardly for the GFP-YAP-Y407F mutant, at the promoters of typical ARE- and TEAD1-driven genes is readily detected but becomes displaced after treatment with J54. (5) While xenografts of LNCaP cells show rapid regression following treatment with ARSI+J54, in the VCaP model, driven by the *TMPRSS2-ERG* oncogenic translocation, tumors initially respond well to the combination but subsequently recur, despite the continuous suppression of pNek1-T141 and pYAP-Y407. This suggests an alternative parallel pathway for CRPC progression for VCaP tumors in the long term, which may be separate from the observed ENZ-driven YAP deregulation, although clearly some YAP gene targets like PD-L1, that are found to accumulate following prolonged ENZ treatment, are still suppressed by the concomitant addition of J54.

## 1. Introduction

The standard of care for advanced prostate cancer (PCa) consists of Androgen Deprivation Therapy (ADT) and the use of ever more effective therapies targeting the androgen receptor (AR) under different mechanisms (reviewed in [1]). Unfortunately, these treatments ultimately fail due to various PCa adaptation pathways [2] resulting in the incurable phase of the disease: metastatic castration-resistant prostate cancer (mCRPC). One of the most common mechanisms is the ability to integrate AR signals with ever-diminishing residual testosterone, with the frequent participation of YAP (the final transducer of the Hippo pathway) as an AR co-activator (reviewed in [3]).

The Hippo pathway responds to a variety of signals, including cell–cell contact, mechanotransduction, and apico-basal polarity [4,5]. When the Hippo pathway is activated, kinases MST1/2 and LATS1/2 phosphorylate and inactivate YAP and TAZ. YAP and TAZ are transcriptional co-activators but lack DNA binding activity. They also appear to lack typical NLS and NES signals [6,7] (although this was recently argued in [8]) and were believed to shuttle via interaction with their transcriptional partners, their association with σ14-3-3 [9], or by the mere cell’s mechanical deformation [10], although a role for YAP post-translation modification has also been proposed [11,12]. Upon phosphorylation by MST and LATS kinases, YAP is sequestered in the cytoplasm, ubiquitylated by the B-TrCP ubiquitin ligase, and marked for degradation by the proteasome. YAP/TAZ are usually inhibited by the cell–cell contact in normal tissues [4]. Over-activation of YAP/TAZ through aberrant regulation of Hippo has been noted in many types of tumors and associated with the acquisition of malignant traits, including resistance to anticancer therapies, the maintenance of cancer stemness, distant metastasis [4], and in prostates, androgen-independent (AI) adenocarcinoma progression [13,14]. When the Hippo core kinases are “off”, YAP/TAZ translocate into the nucleus, bind to TEAD1-4, and activate the transcription of downstream target genes, leading to multiple oncogenic activities, including loss of contact inhibition, cell proliferation, epithelial–mesenchymal transition, and resistance to apoptosis. In PCa, YAP has been identified as a binding partner of AR and colocalized with AR in an androgen-dependent (AD) manner and in an AI manner in CRPC [13]. YAP was also found to be upregulated in LNCaP-C42 cells, and, when expressed ectopically in LNCaP, it activated AR signaling and conferred castration resistance, motility, and invasion (reviewed in [3]). Knockdown of YAP greatly reduced the rates of the migration and invasion of LNCaP, and YAP-activated AR signaling was sufficient to promote LNCaP cells from an AS to an AI state in vitro, and castration resistance in vivo [15]. It also was recently determined that ERG (and the common *TMPRSS2-ERG* rearrangement) activates the transcriptional program regulated by YAP1 and that prostate-specific activation of either ERG or YAP1 in mice induces similar transcriptional changes and results in age-related prostate tumors [16].

However, it has remained unclear what the activators of the Hippo/YAP in PCa are, but we have recently shown preliminary results that TLK1 has an important role in this via activation and induced stabilization, or possibly nuclear relocalization, via phosphorylation by Nek1 at YAP-Y407 [17,18]. We are now solidifying this work by showing the following: (1) The phosphorylation of Y407 is critical for YAP retention/partition in the nuclei, and J54 (TLK1i) reverses this along with YAP407 dephosphorylation. (2) The enhanced degradation of (cytoplasmic) YAP is increased by J54 even when treatment with Enzalutamide (ENZ) tends to lead to its accumulation. (3) The basis for all these effects, including YAP nuclear retention, can be explained by the stronger association of pYAP-Y407 with its transcriptional co-activators, AR and TEAD1. (4) ChIP for GFP-YAP-wt, but much less for the GFP-YAP-Y407F mutant, at the promoters of typical ARE- and TEAD1-driven genes is readily showing occupancy but becomes displaced after treatment with J54. (5) In the VCaP model, driven by the *TMPRSS2-ERG* oncogenic translocation, xenografts initially respond well to the combination of Enzalutamide (ENZ) and J54 (TLKi), but ultimately about 50% of the tumors relapse, despite the fact that J54 is still capable of suppressing pNek1-T141, its target, pYAP-Y407, and some of its target gene products.

## 2. Materials and Methods

### 2.1. Cell Culture Products

An RPMI-1640 medium was acquired from Thermofisher (Norristown, PA, USA). ENZ was acquired from BioSci Inc. (Phoenix, AZ, USA), and J54 was synthesized by our group as described in the STAR Methods of [19]. We, however, also purchased some quantities from MedKoo Biosciences (Durham, NC, USA) (J-3-54).

### 2.2. Antibodies

The following antibodies were used in this study: mouse anti-YAP (dilution—1:1000 in 5% Milk+TBST, Santa Cruz Biotechnology, SCBT, Dallas, TX, USA, cat# sc101199), rabbit anti-phospho-YAP-Y407 (custom generated by Life technologies, Carlsbad, CA, USA), mouse anti-NEK1 (1:1000 in TBST, SCBT, cat# sc 398813, Dallas, TX, USA), rabbit anti-phospho-NEK1 pT141 (lab-generated by Life technologies, Carlsbad, CA, USA), anti-Hu CD274 (PD-L1, B7-H1) (1:1000 in TBST, cat# 14-5983-82, Thermo Fisher, Waltham, MA, USA), anti-ORC2 (1:1000 in TBST, LS-C170256, Shirley, MA, USA), anti-pSTAT1 (1:1000 in TBST, cat# 9167 CST Danvers, MA, USA), anti-pIRF3 (1:1000 in TBST, cat# 4302 CST Danvers, MA, USA), rabbit anti-TEAD1 (1:1000 in TBST, cat# 12,292 CST Danvers, MA, USA), rabbit anti-pSTING (1:1000 in TBST, cat# 19,781 CST Danvers, MA, USA), rabbit anti-STING (1:1000 in TBST, cat# 80,231 CST Danvers, MA, USA), anti-GAPDH (1:1300 in 5%BSA+TBST, ca# 2118S (14C10), CST), anti-GFP (cat# MA5-15256 (GF28R), Thermo Fisher, Waltham, MA, USA), rabbit anti-MMP10 (1:1000 in TBST, Abcam, Cambridge, MA, USA, cat# ab38930), rabbit anti-MMP9 (1:1000 in TBST, Abcam, Cambridge, MA, USA, cat# ab283575), and rabbit anti-actin (1:1000 in TBST, Abcam, Cambridge, MA, USA, cat# ab1801). Secondary HRP-conjugated antibodies, anti-rabbit (cat# 7074S, CST) and anti-mouse (cat# 7076S, CST), were used to probe immunoblots.

#### 2.2.1. GFP Localization

GFP-YAP-expressing cells were seeded at a density of 20,000/cm^2^ in a T25 and after treatment with fresh medium containing J54 (10 µM) were immediately monitored during a time course in the IncuCyte.

#### 2.2.2. ChIP and coIP

These were carried out as described in [20]. The sequence of primers for the respective genes used for the ChIP experiment is listed in Table 1.

### 2.3. Animal Studies

All animals used in this study received humane care based on the recommendations set by the American Veterinary Medical Association, and the Institutional Animal Care and Use Committee of LSU Health Sciences Center at Shreveport approved all the test protocols. Immune-deficient NOD SCID mice (Charles River) were used in this research to host human VCaP tumors. The mice were subcutaneously implanted with 0.5 × 10^6^ human PCa VCaP-Luc cells suspended in Matrigel into each lower back flank. Following establishing tumor sizes less than 120 mm^3^, the mice harboring the tumors were randomly assigned to three groups for treatment. Enzalutamide (Enz, 10 mg/kg), an androgen receptor (AR) inhibitor, and a combination of Enz and the TLK1-NEK1 axis inhibitor J54 (10 mg/kg each) were administered to the mice as treatments. One group received the combination treatment orally (OR). J54 and Enz were administered every two weeks, dissolved in 200 sterile saline containing 10% Polysorbate-80—PS-80. Our prior work served as the basis for the dosage for J54. A caliper was used every other day to measure the tumor diameters. Thirteen biweekly medication cycles totaling forty-three days were spent on the inhibitor therapy. The tumor-bearing mice were observed every other day and were euthanized if their body weight appeared to have dropped by more than 20%, if they showed signs of poor health or stomach palpitations brought on by cancer metastases or prostate tumor development, or if they were too unwell to eat or drink. After the experiment, the mice were sacrificed by CO_2_ asphyxiation, and the tumors were removed to perform tissue Western blots.

### 2.4. Western Blots

#### 2.4.1. Tissue Western Blot

Western blots were performed from randomly selected tumors excised from the different treatment groups, including control (PBS), ENZ, J54, and the combination of the VCaP-Luc grafted NOD SCID mice. The frozen tumor tissues were disrupted with the Bioruptor^®^ Plus sonication device (Diagenode, Denville, NJ, USA; Cat. No. B01020001), homogenized, and lysed in the ice-cold RIPA lysis buffer system (Santa Cruz Biotechnology; Cat. No. SC-24948). The samples were clarified by centrifugation at 13,000 rpm for 20 min in the refrigerated setting. The supernatant was collected, transferred into fresh 1.5 mL microfuge tubes, flash-frozen, and stored at −80 °C until further use. The total protein concentration was measured using a Pierce™ BCA protein assay kit (Thermo Scientific; Cat. No. 23225) with bovine serum albumin (BSA) as a standard control. An equal loading amount of 15 µg was calculated for each protein sample. The sample supernatant was denatured with 1X Laemmli Buffer for 10 min at 95 °C and separated using 10% PAGE with Mini PROTEAN TGX protein gel (BioRad, Hercules, CA, USA; Cat. No. 4568084) at 100 volts for 120 min. The proteins were transferred to the Immun-Blot PVDF membrane (BioRad; Cat. No. 1620177) using a Mini Trans-Blot Cell (BioRad; Cat. No. 1703930) at 100 volts for 150–180 min on ice. The membrane was blocked with 5% non-fat dry milk (Cell Signaling Technology, Danvers, MA, USA; Cat. No. 9999S) in 1X Tris-buffered saline with Tween-20 (TBST) for 1 h at room temperature. Following blocking, the membrane was washed once with 1X TBST and incubated with primary antibodies in 5% BSA in 1X TBST overnight at 4 °C with gentle rocking. The next day, after washing four times with 1X TBST, the membrane was incubated with horse anti-rabbit antibody (1:2000 dilution) labeled with horseradish peroxidase in 5% BSA in 1X TBST for 1–1.5 h at room temperature. After incubation, the membrane was washed four times with 1X TBST, and the reactive bands were detected using Pierce™ ECL Western Blotting Substrate (Thermo Scientific; Cat. No. 32106) on ChemiDoc MP Imaging System (BioRad; Cat. No. 12003154).

#### 2.4.2. Cell Western Blot

The Western blot for PC-3 cells was performed as described above but with minor modifications. Briefly, 3 × 10^6^ LNCaP-expressing GFP-YAP or VCaP cells (control and drug-treated) were collected, washed twice with ice-cold PBS, and lysed with RIPA lysis buffer system. The lysate was vortexed and centrifuged at 13,000 rpm for 10 min to remove cell debris. The total protein was estimated, and 30 μg of the cell lysate was loaded onto an SDS-PAGE gel. The separated proteins were transferred to the membrane using a wet transfer apparatus. The complete transfer was ensured by checking the membrane for uniform background staining. The membrane was then incubated in a blocking solution (e.g., 5% non-fat milk in TBST) for 1 h at room temperature to block non-specific binding sites, followed by a primary antibody.

### 2.5. Statistical Analysis

Statistical analyses were performed using Graphpad prism 9 and Microsoft Excel software (Version 16.88). Data quantifications are expressed as mean ± standard error of the mean (SEM). Statistical significance was calculated by a 2-tailed Student’s *t*-test when comparing the mean between two groups, or by a one-way ANOVA followed by Tukey’s post hoc analysis when comparing more than two groups. *p*-values  <  0.05 were considered significant.

## 3. Results

### 3.1. The TLK1>Nek1 Nexus Is a Key Player of YAP Stability

We previously showed that activation of the TLK1>Nek1 nexus results in an increased expression of YAP, largely via the nexus leading to the accumulation of this typically rapidly degraded protein, and Nek1-KO cells have intrinsically very low levels of YAP [17]. We subsequently showed that the Y407F mutant is expressed in LNCaP and Hek293 cells as a highly unstable protein, constitutively displaying an array of cleaved smaller species, and reduced transcriptional activity [18]. However, it is unknown if the majority of CRPC progression journeys through the overactivation of YAP [21], and if so, what the key regulators of the Hippo/YAP axis in PCa are.

**Figure 1:**
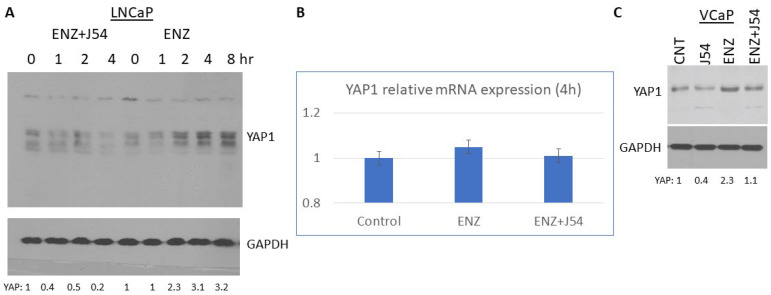
YAP-increased expression in LNCaP treated with ENZ is suppressed with J54. (**A**) LNCaP cells grown in 6-well plates were treated with ENZ+/−J54 (1 µM each) for indicated times. Cell lysates (20 µg) were processed for WB for YAP and mRNA expression (**B**). (**C**) VCaP cells grown in 6-well plates were treated with ENZ+/−J54 (1 µM each) for 4 h, and cell lysates were thereafter processed for WB. The uncropped bolts are shown in Supplementary Materials.

Relatively few PCa cell models exist to monitor the adaptive changes that occur during the progression from AS to AI growth, with the predominant one clearly being the LNCaP cells. With these cells, it was demonstrated the critical importance of YAP1 and its integration of AR and TEAD signals reprogramming in their transition to ENZ resistance [22], which was also correlated to functional analyses revealing that YAP1 positively regulates numerous genes related to cancer stemness and lipid metabolism. Moreover, gene signatures of COUP-TFII and YAP1 from different datasets showed a strong correlation in a large cohort of mCRPC datasets from cBioPortal [22]. However, in that study, the expression of YAP1 was analyzed in LNCaP clones derived for their resistance to ENZ (EnzaR) and not by following early stages of adaptation to ARSI exposure. In fact, we have recently shown that YAP1-increased expression following ADT is largely post-transcriptional, and due to the rapid compensatory activation of the mTOR>TLK1B>NEK1 kinase cascade, it ends in the phosphorylation of YAP1-Y407 that transcriptionally activates as well as prominently stabilizes this key co-activator [17,18]. We now show that this is in fact a rapid event following exposure of LNCaP cells to ENZ, and it is possibly an important survival mechanism to integrate any residual AR signals (or other YAP1 signatures) that lead to their final adaptation to ARSI exposure. We show that the rapid increase in YAP1 accumulation after can be completely blocked by concomitant treatment with J54 (Figure 1) without appreciable changes in the mRNA, which was thus attributed to YAP1 protein stabilization [17,18], although the newly uncovered Nek1>YAP regulation is underreported in recent reviews [23]. Nonetheless, we established that activation of TLK1 by BIC and the resulting increase in pNek1-T141 and transcriptional activity [18] can be completely suppressed with J54 (TLKi) [24] as a direct regulator of YAP stability. This could also be demonstrated in VCaP cells treated for 4 h as shown (Figure 1C), which represents another rare PCa model that readily converts in culture and in xenografts from AD to AI following ARSI treatment.

While Figure 1 can invoke possible alternative explanations for the progressive difference in YAP levels in ENZ- vs. ENZ+J54-treated cells over time, this result is independently reinforced by our prior report that GFP-tagged versions of YAP showed an increased pattern of cleaved products after treatment with J54 for the wt version, whereas the Y407F mutant showed constitutively high instability and degradation products that were not enhanced after treatment with J54 (as the key Nek1 phosphorylation site was abrogated) [18].

### 3.2. Evidence That TLK1>Nek1>pYAP-Y407 Is Critically Important for Its Nuclear Localization/Retention

We previously reported that GFP-YAP-wt appeared to be preferentially localized to the nuclei, whereas the Y407F mutant seemed largely excluded from the nuclear compartment [18]. However, that was only a suggestion, as the cells had not been sorted for GFP expression and rather just selected for G418 resistance. Moreover, we had not tested the dependence on the TLK1>Nek1 nexus by challenging the cultures with J54.

**Figure 2:**
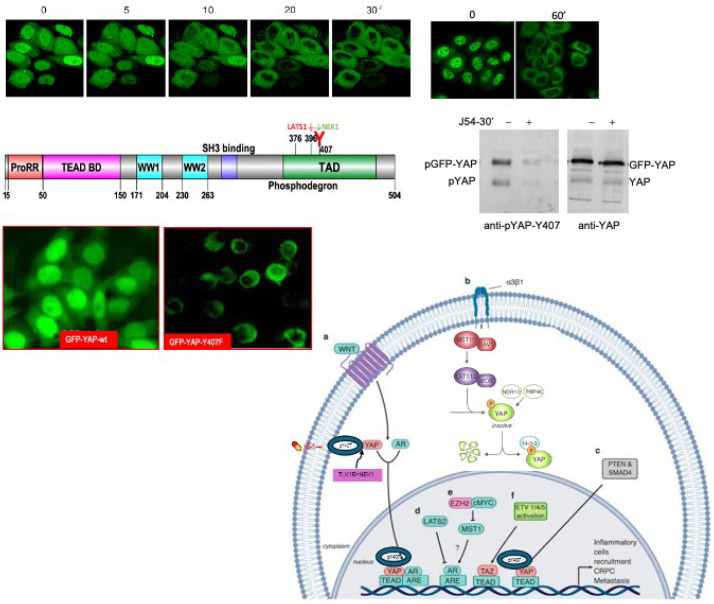
J54 elicits rapid GFP-YAP-Y407 dephosphorylation and nuclear export prior to cytoplasmic degradation—the default of an active Hippo pathway (LATS1-mediated pS396). Microscopic and WB depiction of the process and a graphical illustration. The uncropped bolts are shown in Supplementary Materials.

We now show exactly that effect in Figure 2, which demonstrates that treatment with J54 results in the rapid dephosphorylation of pYAP-Y407 (GFP-tagged or endogenous protein) and concomitant relocalization from the nuclei to the cytoplasm. We also confirmed more rigorously that GFP-YAP-wt is mostly nuclear, whereas the Y407F mutant is largely excluded from the nuclei via cellular fractionation (below). A working model is also shown in Figure 2 (adapted from [3]) where we propose that the productive association of pYAP-Y407 with transcriptional co-activators leads to its import and/or retention in the nuclei and at promoters of its target genes, whereas J54-mediated dephosphorylation leads to its default: LATS-mediated phosphorylation at S127 and S396 and subsequent nuclear export via shuttling with σ14-3-3 proteins and eventually its rapid degradation.

### 3.3. The TLK1>Nek1 Nexus Is Actively Involved in the Regulation of the Nucleocytoplasmic Shuttling of YAP

**Figure 3:**
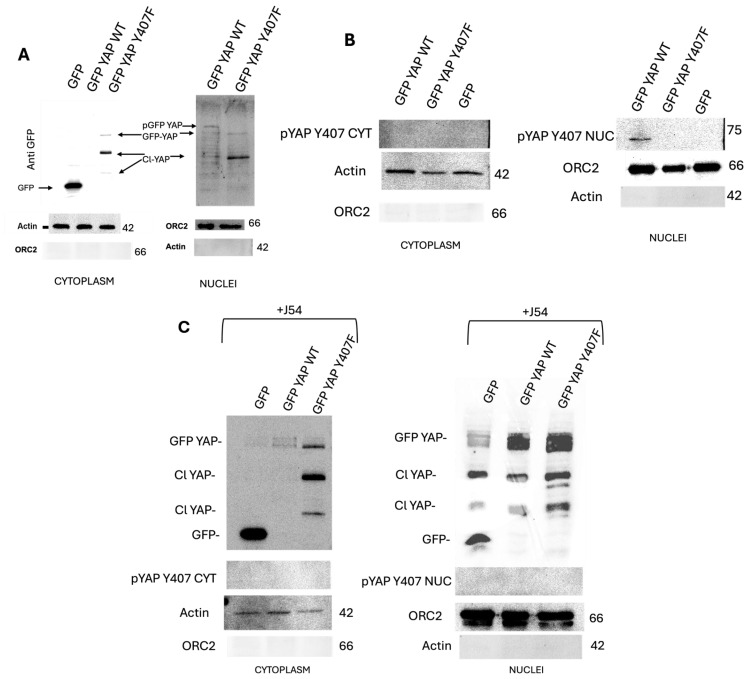
Cell fractionation reveals nuclear localization of GFP-YAP-wt, and it is predominantly cytoplasmic for the Y407F mutant. (**A**) shows the subcellular localization of GFP YAP when the cells were probed with anti-GFP while (**B**) depicts the localization of the pYAP Y407 in the respective cells. (**C**) Subcellular redistribution YAP and pYAP Y407 upon treatment with J54 (a TLKi). Actin was used as a marker for the cytoplasmic fraction and was absent in the nuclei. Orc2 was used as a marker for the nuclei and was not present in the cytoplasm even when the blot was overexposed to reveal some cross-reacting bands known to be detected with this SL-Bio antiserum (see Supplementary Materials).

The subcellular location of YAP is pertinent for its downstream signaling, and several studies have confirmed that active YAP must be essentially nuclear to facilitate its transcriptional programs [25,26]. We previously reported that LNCaP cells overexpressing GFP-YAP-WT and not the Y407F mutant can convert from AS to AI growth. We now provide evidence to show that this transformation is driven by the partial relocation of YAP to the nucleus after Nek1-mediated phosphorylation (Figure 3A) and that the Y407F mutant’s high instability and degradation in both the nucleus and the cytoplasm could explain its inability to transform cells into AI growth. This property highlights the need for the spatiotemporal regulation of YAP activity via nucleocytoplasmic shuttling. In response to the numerous cues that signal through the Hippo pathway, phosphorylation and dephosphorylation of YAP, by its core kinases, have been reported to regulate its cytoplasmic and nuclear movements, respectively [27]. A recent report of the involvement of Src-family kinases in the shuttling process by [28] suggests the participation of other YAP nucleocytoplasmic regulators independent of Hippo core kinases. We now report that Y407 phosphorylation of YAP is sufficient for relocating active YAP into the nucleus (Figure 3B), as evident by the YAP-Y407 band seen only in the GFP-YAP-WT, where it can stably bind to DNA-binding partners to initiate the transcription of target genes. (Figure 3C). The less interactive YAP-Y407F is shuttled out from the nucleus likely associated with the σ14-3-3 proteins and into the cytoplasm where, in time, it is targeted for degradation by the b-TrCP, the default of Hippo core kinase phosphorylation.

### 3.4. GFP-YAP-wt Partitions Largely to the Nuclei Whereas the Y407F Mutant Partitions to the Cytoplasm

A fractionation experiment to separate nuclei from cytoplasm revealed the following: GFP-YAP-wt is mostly nuclear (with a clearly slower mobility band, seen also in C, enriched in the nuclear fraction) and absent from the cytoplasm (Figure 3A) whereas the GFP-YAP-Y407 mutant distributes about 60:40 between the cytoplasm and the nuclei (note also the prominent degradation product (Cl-YAP) that we previously reported [18] and its presence in both cytoplasm and nuclei). When we used the phospho-specific pY407 antiserum (Figure 3B), the only signal seen was from the WB of the GFP-YAP-wt cells, in the nuclear fraction. However, phosphorylation of Y407 is not absolutely required for YAP nuclear localization. Figure 3C shows that regardless of the use of J54 or of the Y407F mutant, some GFP-YAP is found in the nuclei when this fraction is enriched. The lower panels in C, which were probed with pYAP-Ab, confirmed that the treatment with J54 abolished its phosphorylation.

### 3.5. Y407 Phosphorylation of YAP Is Important for Its Nuclear Interaction with DNA-Binding Partners and Delivery of Transcriptional Outputs That Control CRPC Progression and ECM Invasiveness

YAP as a transcriptional co-activator lacks a DNA-binding domain and hence requires a DNA-binding transcription factor to modulate the expression of its target genes [29]. In addition, its association with these binding partners has been suggested to be beneficial for its nuclear retention [9]. Although YAP interacts with numerous DNA-binding transcription factors [29,30], the TEAD family of proteins remains the most characterized binding partner of YAP. The interaction with TEAD is significant given that a sizable proportion of the N-terminal of YAP is devoted to TEAD binding [31], and the nature of this interaction is often described as pro-oncogenic [32]. Specifically, in PCa, YAP has also been described to co-localize with AR in the androgen-dependent and AI manner to form protein complexes in the nuclei [13].

**Figure 4:**
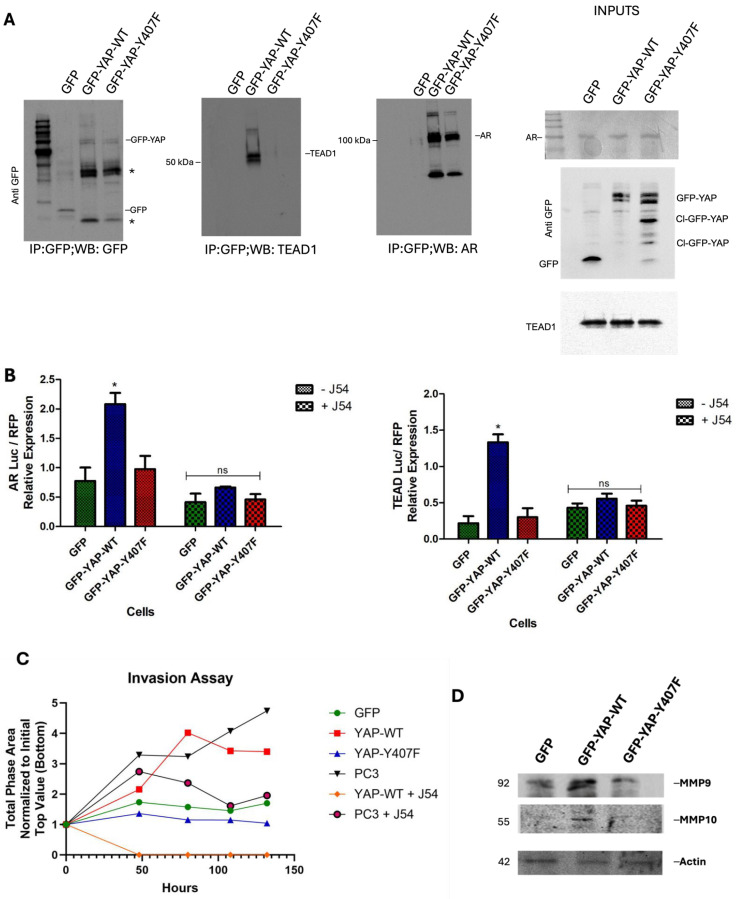
Stronger association of GFP-YAP-wt with its transcriptional co-activators. (**A**) IPs were carried out with GFP antiserum, and WBs were probed for GFP, TEAD1, or AR. Inputs are also shown in the right panel. (**B**) A luciferase reporter assay showing the stronger association of the YAP-WT and its reversal with J54 treatment. (**C**) The Matrigel invasion assay reveals the invasive property of the respective cells and the effect of J54 treatment on YAP-WT’s invasive potential. (**D**) The immunoblot for MMPs ascertains the involvement of MMP9 and MMP10 for basement invasion. The uncropped bolts are shown in Supplementary Materials. * Significant as *p* > 0.01.

We previously reported the transcriptional output from Y407 phosphorylation in an expression panel of EMT and AR target genes [18]. To elucidate the importance of Y407 phosphorylation of YAP in converting upstream signaling to transcriptional outputs in LNCaP cells, we carried out co-immunoprecipitation with GFP-YAP-WT and the Y407F-mutant-expressing cells. Our results revealed that wt-YAP interacts much more strongly with TEAD and AR compared to the mutant (Figure 4A).

Moreover, using the transcriptional reporters of AR and YAP/TEAD (8xGTIIG-Lux) in a transient transfection experiment, we also confirmed that the transcriptional output of wt-YAP is significantly higher than that of the mutant following Nek1>YAP1 activation (Figure 4B), which supports the upregulation of the AR target, and YAP/TEAD canonical genes. Interestingly, we observed that the J54 treatment resulted in a significant reduction in the luciferase expression to a level comparable to the mutant when both AR and TEAD reporters were considered. This rightly connotes that J54, and by extension the obliteration of Y407 phosphorylation, also interrupts YAP’s interaction with binding partners in addition to disrupting its stability. In [18], we confirmed that Y407 phosphorylation is critical for EMT, and we further investigated the implications of YAP’s stable association with binding partners on its tumor-invasive properties using the IncuCyte cell migration assay. We observed that LNCaP cells (typically rather indolent) overexpressing YAP-wt had a high basement membrane (Matrigel) invasive ability (Figure 4C) comparable to that of the highly metastatic PC3-PCa cells. This effect was reversed with the J54 treatment of the cells, again depicting the ability of J54 to reverse the metastatic properties of PCa.

We carried out an immunoblot to determine the expression of some of the known MMPs and found a higher expression of MMP9 and MMP10 (Figure 4D) in the wt-YAP-overexpressing cells which corresponds with an increased YAP activation of EMT TFs which we have previously described in [18].

### 3.6. YAP-Y407 Phosphorylation Promotes Interaction with Cis-Regulatory Elements at the Promoters of Target Genes

**Figure 5:**
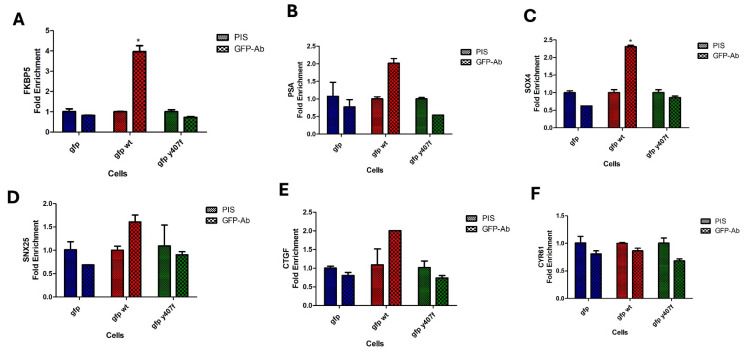
ChIP of GFP-YAP-wt vs. Y407F mutant at promoters of canonical CRE and ARE target genes reveals significantly different occupancy. PIS is pre-immune serum vs. GFP antiserum. GFP-YAP-WT increasingly occupied promoters of (**A**) FKBP5, (**B**) PSA, (**C**) SOX4, (**D**) SNX25, (**E**) CTGF, and (**F**) CYR61 genes compared to Y407F mutant. * Significant as *p* > 0.01.

We have shown that Y407 phosphorylation of YAP greatly enhances its strong interaction with TEAD which will provide a means to contact the DNA indirectly for successful downstream responses. With the presence of TEAD consensus motifs on promoters and enhancers of several YAP/TAZ-bound cis-regulatory elements (CREs) [19,33], YAP could mediate oncogenesis by indirect interaction with CREs at these promoters. In fact, it was reported in [19] that such a YAP-mediated CRE interaction is a driver of colorectal cancer. Emerging evidence suggests that the YAP/TEAD complex also recruits other transcription factors to the active enhancer or promoter of specific genes [34]. We investigated how Y407 phosphorylation of YAP could influence its interaction with CRE at the promoters of selected YAP/TEAD and AR target genes via chromatin immunoprecipitation. We observed increased promoter occupancy of wt-GFP-YAP, but much less for the Y407F mutant, at the AR target genes, FKBP5 (Figure 5A), PSA (Figure 5B), SOX4 (Figure 5C), and SNX25 (Figure 5D), as well as the YAP/TEAD target genes, CTCF (Figure 5E) and CYR61 (Figure 5F), compared to the mutant (Figure 5A) which further supports the notion that Y407 phosphorylation of the YAP not only stabilizes YAP but also increases its association with TFs and its colocalization of a bridged complex on the CREs of the promoters of target genes. This eventually could lead to the transcription of genes involved in oncogenic growth and/or CRPC progression.

### 3.7. Treatment of VCaP-Xenografted Mice with ENZ and J54 Shows Only Transient Tumor Regression Despite Prolonged Suppression of TLK1>NEK1>YAP Axis

**Figure 6:**
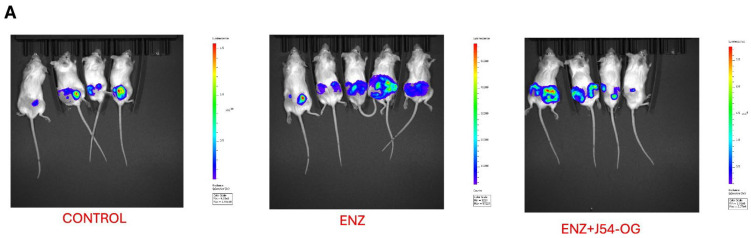

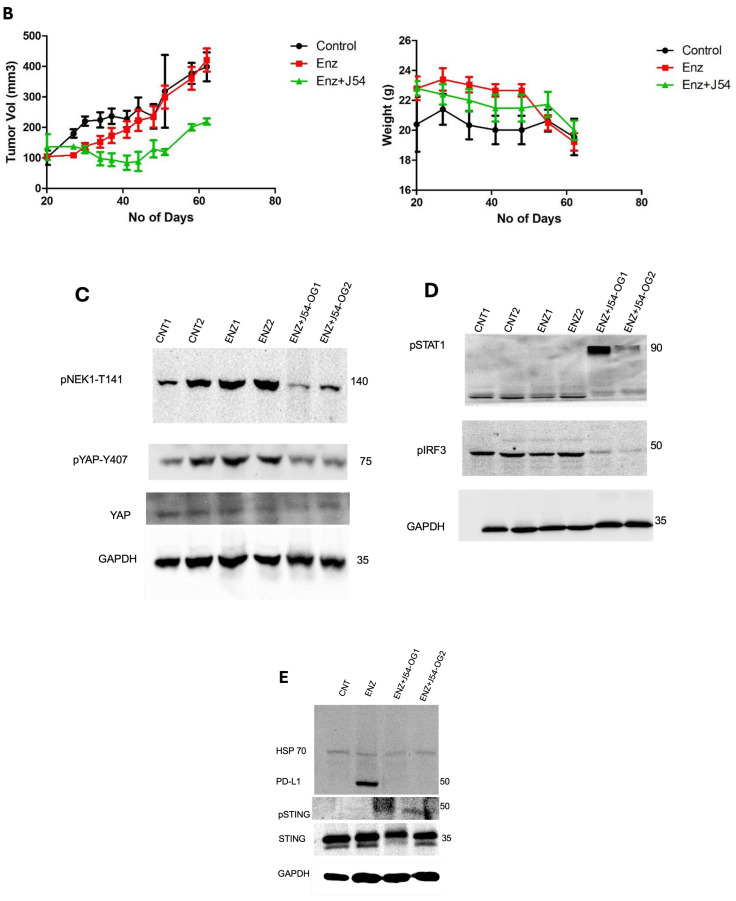
Treatment of mice harboring VCaP subcutaneous flank tumors. (**A**,**B**) After inoculation of 10^6^ cells in each flank of NOD-SCID mice, treatment started when the tumors reached 120 mm^3^, and resulted in a brief growth suppression with ENZ alone and was more sustained in combination with J54, but after ~2 months ((**A**)–end-point), most tumors relapsed and were processed for multi-panel WBs (**C**–**E**). Note that pNek1-T141 and pYAP-Y407 (**C**) were generally increased in animals treated with ENZ but suppressed when concomitantly treated with J54. Total YAP was slightly decreased with J54. The uncropped bolts are shown in Supplementary Materials.

We previously reported that treatment of LNCaP xenografts with ARSI+J54 results in a nearly complete, permanent regression of tumors [24,35]. The VCaP xenograft model, which also converts readily from AD to AI, initially responds well (with shrinkage of the tumors) to the combination of Enzalutamide (ENZ) and J54 (TLKi), but ultimately in about 50% of the mice, the tumors slowly re-grow (Figure 5A,B). This is despite the fact that J54 is still capable of suppressing pNek1-T141 and its target, pYAP-Y407 [18] (Figure 5C), the active, nuclear form of the co-activator, and some of its target genes that encompass immune checkpoint regulators (Figure 5D,E). We currently favor the hypothesis that the *TMPRSS2-ERG* oncogenic translocation expressed in VCaP can implement a parallel AI (CRPC) conversion as the one mediated by the AR/YAP integration, with elevated expression of their target genes including EMT and androgen independence determinants [18], but that the addition of J54 to curtail the TLK1>Nek1 axis may still act as an ICB [36,37] in an immunocompetent host/individual. In fact, an important distinction that characterizes YAP-co-opted regulation of cancer progression genes is its capacity to mediate YAP-induced PD-L1 expression that drives immune evasion [21,35,36]. This is clearly shown in Figure 6E, which shows that prolonged treatment with ENZ results in a dramatic increase in PD-L1 expression, which can be fully suppressed by the concomitant administration of J54. It is noteworthy that this is a sad outcome already reported for ENZ that may limit its prolonged use in patients [37]. Concomitantly, pIRF3 in recidivate VCaP xenografts was also suppressed with J54 (Figure 6D), thus curtailing a key branch of the innate immunity through the expression of INF-γ that is also critical for PDL-1/2 expression and avoiding immune surveillance [38], while pSTAT1 was dramatically increased likely through the activation of cGAS/STING from the release of nuclear and mitochondrial DNA upon implementation of apoptosis [39,40,41,42]. This was confirmed by WB analysis of pSTING vs. pan-STING (Figure 6E), an indication of the high rate of apoptotic turnover in the tumors of mice concomitantly treated with J54. There was clearly variability in the extent of pSTING, as the activation of the cGAS/STING pathway will depend largely on the apoptotic drivers experienced in each tumor, which will depend on various parameters like the TME and hypoxic conditions. Regardless of this variability, J54 strongly enhances these conditions and results in (variably) increased pSTING. Note that in this experiment there is no J54 alone group, which in previous work we had already shown has a negligible effect on the rate of tumor growth or the general health of the mice [24,43].

## 4. Discussion

YAP1 and its paralog TAZ are the final effectors of the Hippo signaling pathway, which is involved in regulating organ size through multiple cellular functions including cell proliferation, differentiation, and apoptosis [3,5,23,44,45,46,47,48]. The Hippo pathway responds to a variety of cellular cues, including cell–cell contact, mechanotransduction, and apico-basal polarity [4,5]. When the Hippo signaling is activated, kinases MST1/2 and LATS1/2 phosphorylate and inactivate YAP1 and TAZ. YAP1 and TAZ are transcriptional co-activators but lack DNA binding activity. Upon phosphorylation by MST and LATS kinases, they are sequestered in the cytoplasm, ubiquitylated by the b-TrCP ubiquitin ligase, and marked for degradation by the proteasome. YAP1/TAZ are usually inhibited by the cell–cell contact in normal tissues [4]. Specifically, it should be noted that YAP1 is a generally unstable protein whose turnover rate is strongly regulated by multiple stabilizing [49] or de-stabilizing phosphorylation events controlled by multiple kinases (see [4,5,15] for some reviews). The best-known destabilizing event is by the Large Tumor Suppressor 1 and 2 (LATS1/2), the core kinases of the Hippo signaling pathway that can phosphorylate YAP1 on Ser127 which creates a binding site for the 14-3-3 protein. The binding of 14-3-3 to pYAP1-S127 leads to its cytoplasmic sequestration [50,51]. Sequential phosphorylation by LATS1/2 on YAP1 Ser397 primes it for further phosphorylation by Casein kinase 1 (CK1δ/ε) on Ser400 and Ser403 which creates a phosphodegron motif in the transcriptional activation domain (TAD) for β-TrCP/SCF E3 ubiquitin ligase-mediated proteasomal degradation [45]. It could be argued that the main regulation of YAP/TAZ is via its translocation from the ‘inactive’ cytoplasmic compartment where it is eventually degraded to its final target genes in the nucleus, and much of this regulation occurs via the phosphorylation of specific residues, e.g., [49,52,53], and importantly, tyrosines [11,49]. The phosphorylation of YAP1 by NEK1 on Y407 (Figure 1A), which is located in the TAD, was a new finding by our lab and immediately correlated with its stabilization since the pharmacologic inhibition of the TLK1>NEK1 nexus with THD or J54 resulted in a dose- and time-dependent degradation of YAP1 (Figure 4B and [17]). Thus, it is notable that the overactivation of YAP1 can be directly suppressed via inhibition of the TLK1>NEK1 activation loop, for example with J54.

We now solidify these initial results by confirming that the phosphorylation of the Y407 residue is critical for the nuclear shuttling or retention of YAP, likely by promoting its association with its transcriptional co-activators. This is vaguely reminiscent of the well-established role of pY containing specific residues as an anchor for SH2-domain proteins [54,55]. The ultimate goal of this study is to identify a pathway for preventing CRPC progression via dampening the TLK1>Nek1>YAP-mediated conversion from AD to AI [56]. We can at this point be confident that this is probably the case for PCa cases that pattern along the LNCaP cell models, but this may not be the case for the more complex situation when the common *TMPRSS2-ERG* translocation is present, and almost certainly not for NEPC cases, wherein the expression of YAP is actually reduced [57] and probably inactive. However, the *TMPRSS2-ERG* oncogenic translocation is known to activate/stabilize YAP [58], consequently driving the expression of PD-L1 [36], which is a critical component of the immune checkpoint and a target for ICB intervention [59]. Notably, parallel work by other investigators has highlighted the important emerging role of the TLKs in this novel area of work [60,61].

## 5. Conclusions

The Hippo pathway, with the ultimate control of the YAP-mediated transcriptional regulation of the genes involved in tumor progression and drug resistance, is largely accomplished through its nuclear/cytoplasmic shuttling. However, the regulators of such relocalization and the mechanisms are still unknown, particularly for PCa. In this work, we demonstrate that TLK1>NEK1. YAP phosphorylation of a key residue (Y407) is critically important for the nuclear retention, transcriptional activation, and stabilization of YAP, supposedly because of its increased interaction with its ultimate co-activators. While suppression of this process with J54-mediated inhibition of pY407 results in complete tumor regression for the LNCaP xenograft model [24], the VCaP xenografts, additionally dependent on the ERG-TMPRSS2 oncogenic translocation, are partly resistant to the combined treatment of ENZ+J54 (which normally results in apoptosis). However, J54 can still mediate suppression in vivo of pNEK1 and pYAP, and consequently, PD-L1, which is known to be upregulated upon prolonged ENZ treatment, likely resulting in the suppression of anti-tumor rejection mechanisms in immunocompetent individuals. Thus, we propose that complementing treatment with ARSI can benefit nonetheless from the addition of J54 to the therapy.

## Figures and Tables

**Table 1:** A list of the primer sequences for the respective genes used for ChIP studies in 5′–3′. F—forward (sense primer), R—reverse (antisense primer).

Gene	Primer Sequence (5′–3′)
KLK3 F	CCA AGT TCA TGC TGT GTG CT
KLK3 R	CCC ATG ACG TGA TAC CTT GA
FKBP5 F	AGC AGC AGG GTG AGG ATG
FKBP5 R	GAC TGC GGC TGT GAA GGT
SNX25 F	GCT CAG ATG ACT ACC TTA GAA AAG CA
SNX25 R	TTA ATC TAG AAC CTC TTA TTC CCA AAC
SOX4 F	CTA TAG GCA GCT CAC AAATGC AA
SOX4 R	ATT TGT AAA GGA ATG CAA TGT TCT GT
CTGF F	TGT GCC AGC TTT TTC AGA CG
CTGF R	TGA GCT GAA TGG AGT CCT ACA CA
CYR61 F	CAC ACA CAA AGG TGC AAT GGA G
CYR61 R	CCG GAG CCC GCC TTT TAT AC

## Data Availability

A description of all data and materials can be found in the referenced article. No additional data have been withheld from the public.

## Supplementary Materials

The following supporting information can be downloaded at https://www.mdpi.com/article/10.3390/cancers16162918/s1, PDF File: Original Western Blot images.
