# Supplementary figures and images for: TLK1>Nek1 Axis Promotes Nuclear Retention and Activation of YAP with Implications for Castration-Resistant Prostate Cancer
Source: Cancers (Basel). 2024 Aug 22;16(16):2918. doi: 10.3390/cancers16162918 (PMC11352418; doi:10.3390/cancers16162918)

Fig. 1

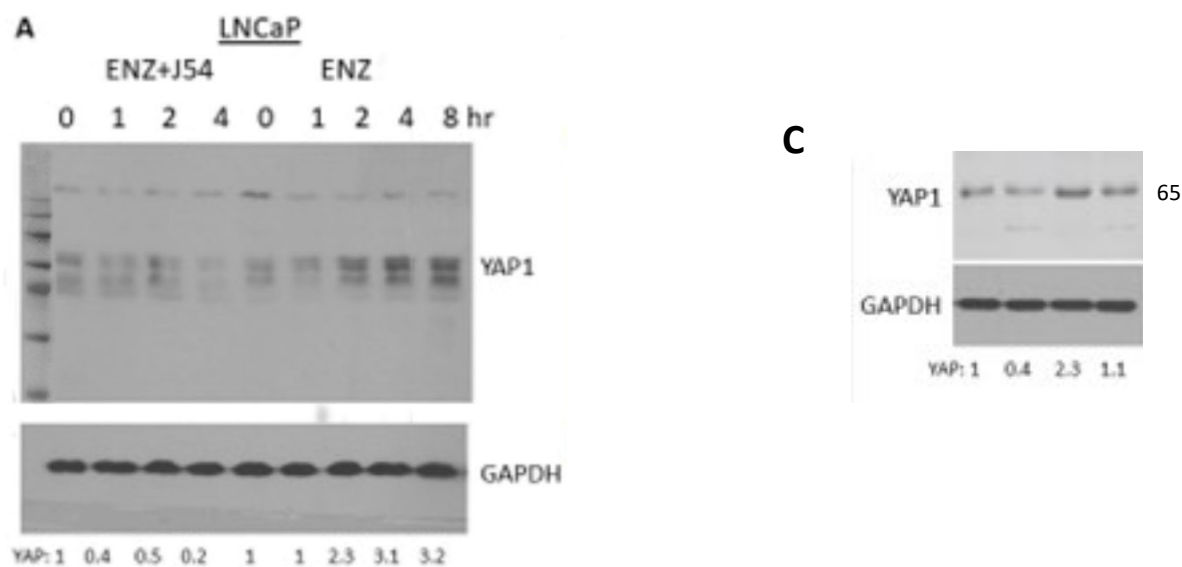

Fig. 2

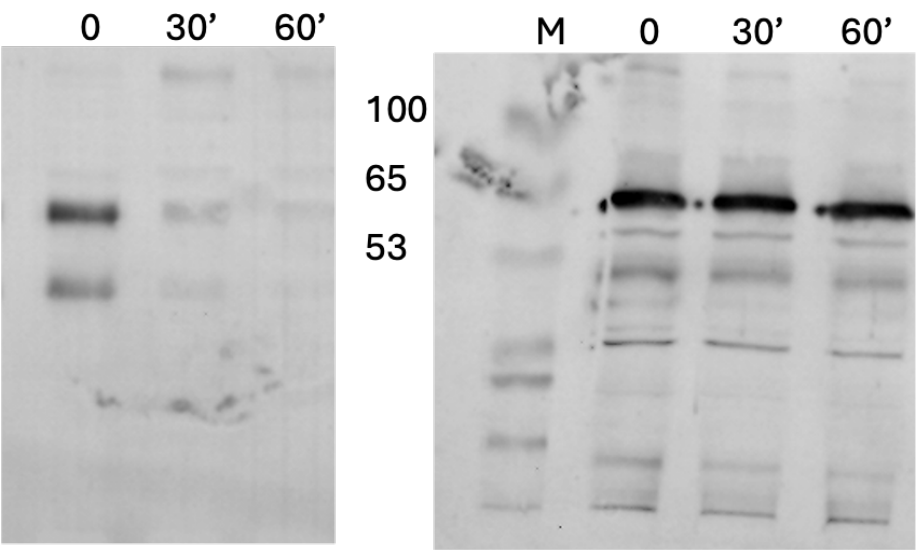

Fig. 3

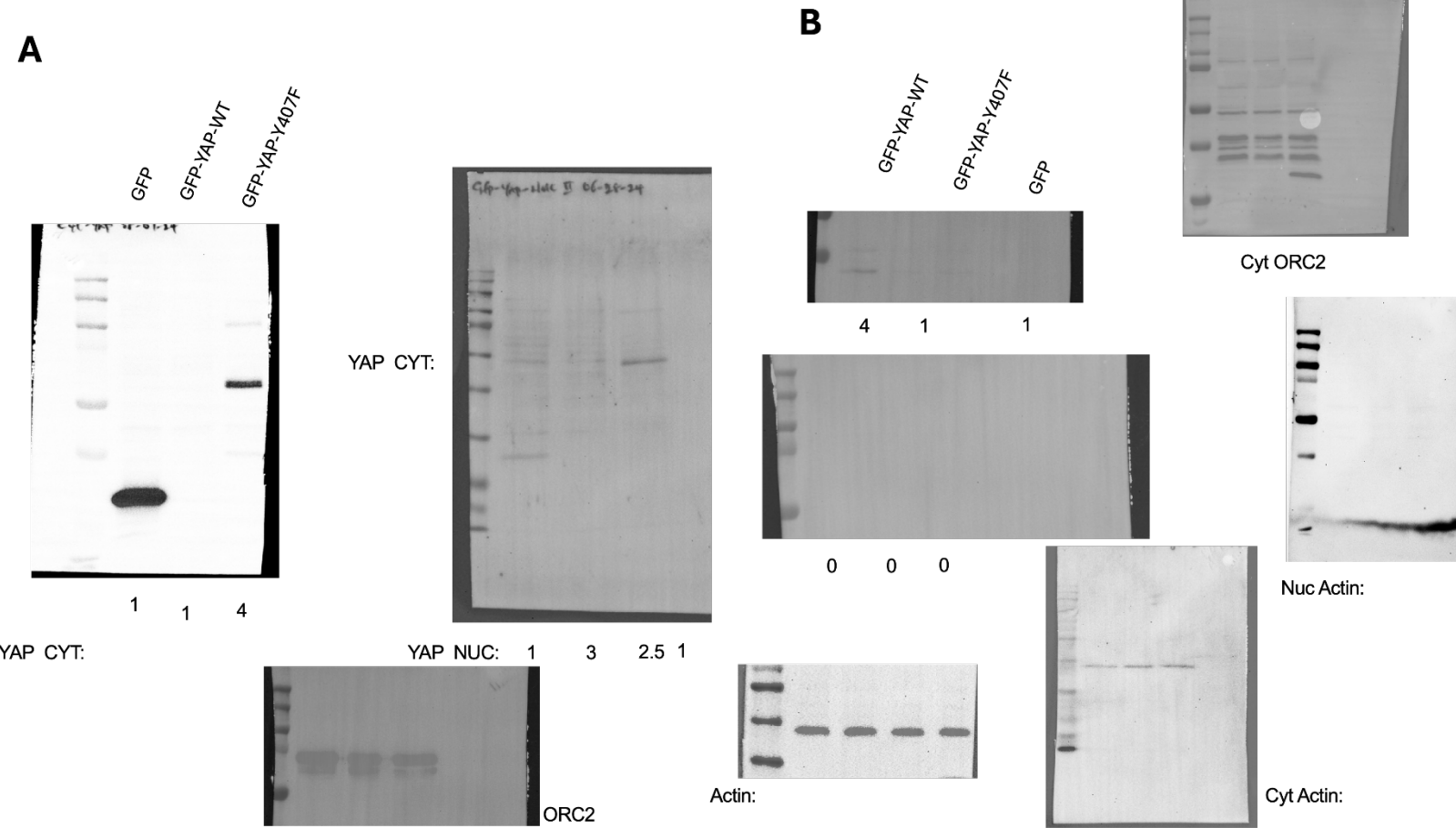

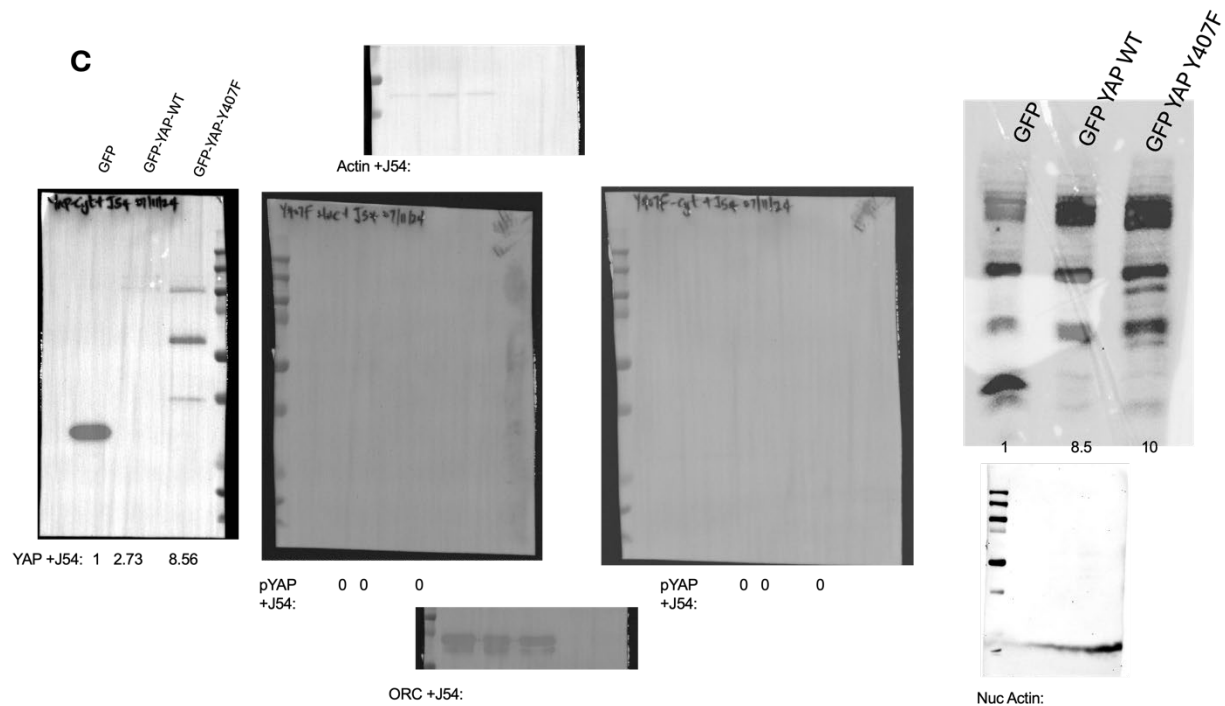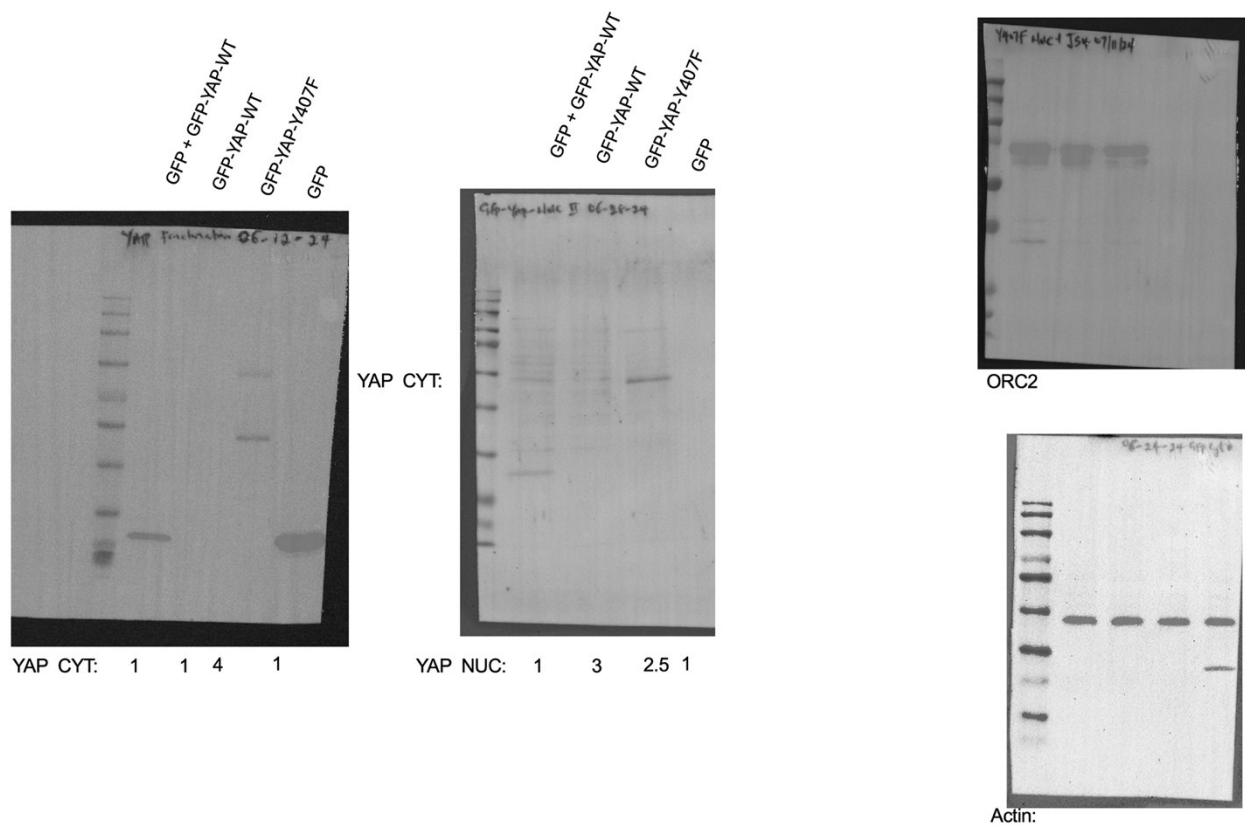

Fig 4

A

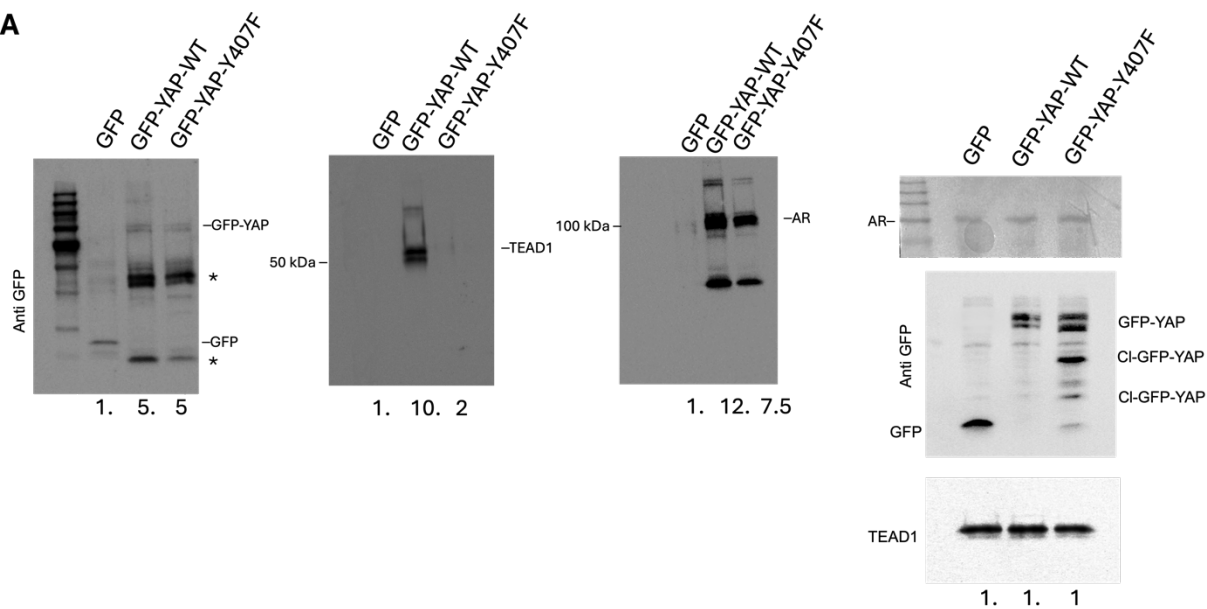

D

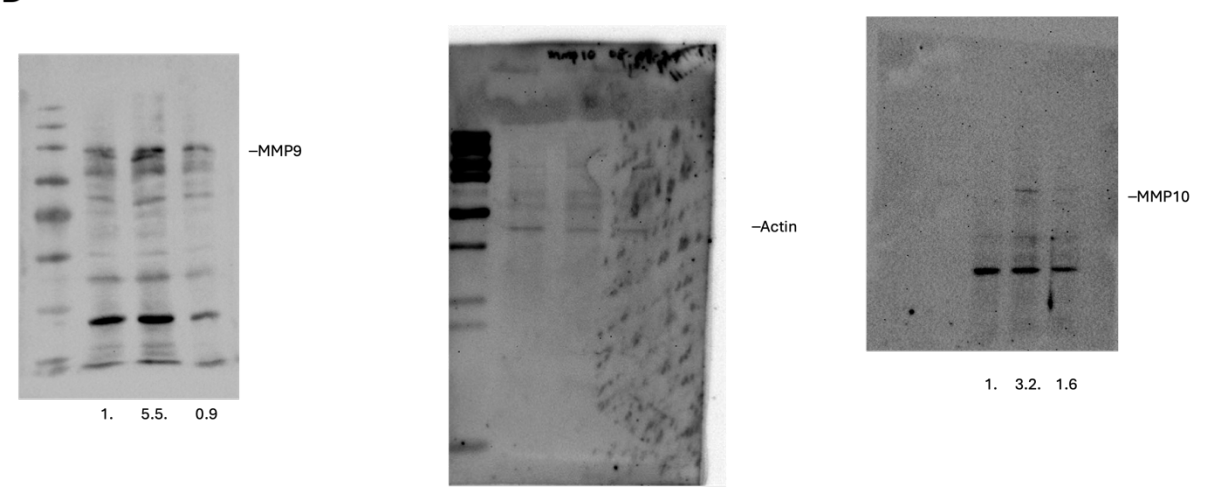

Fig 6

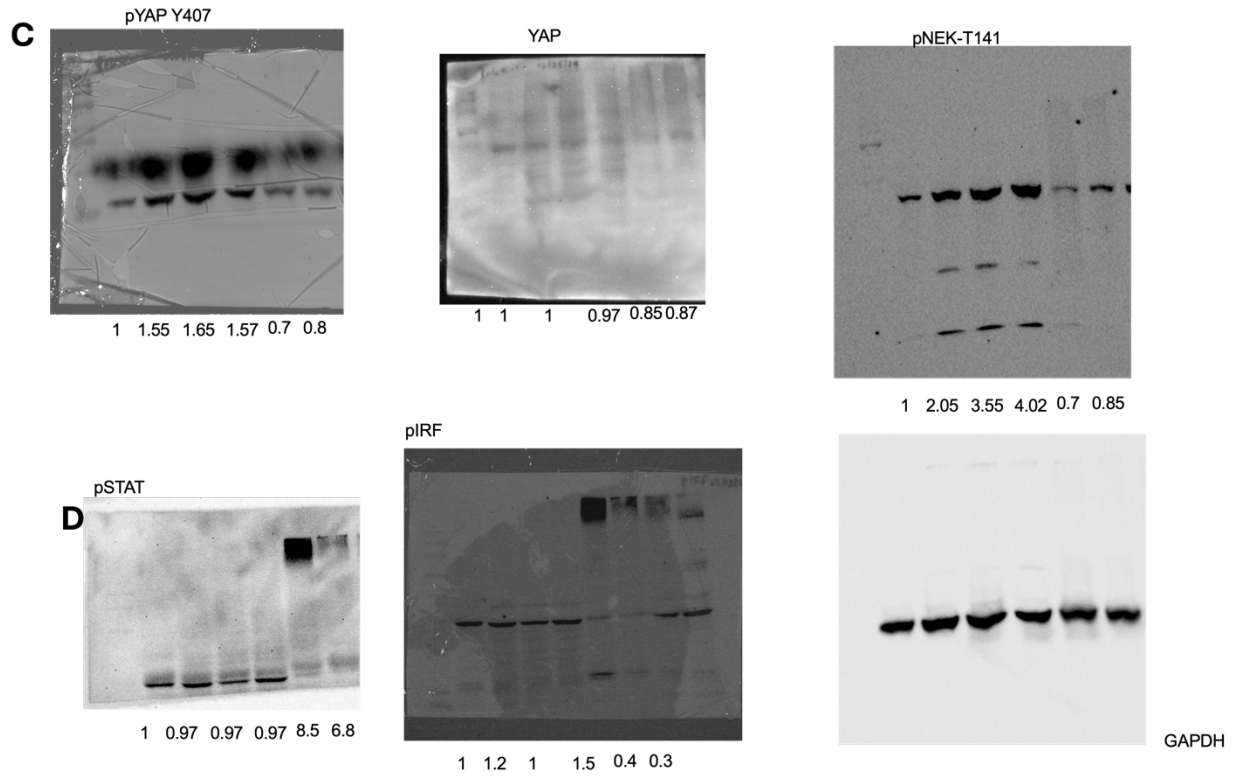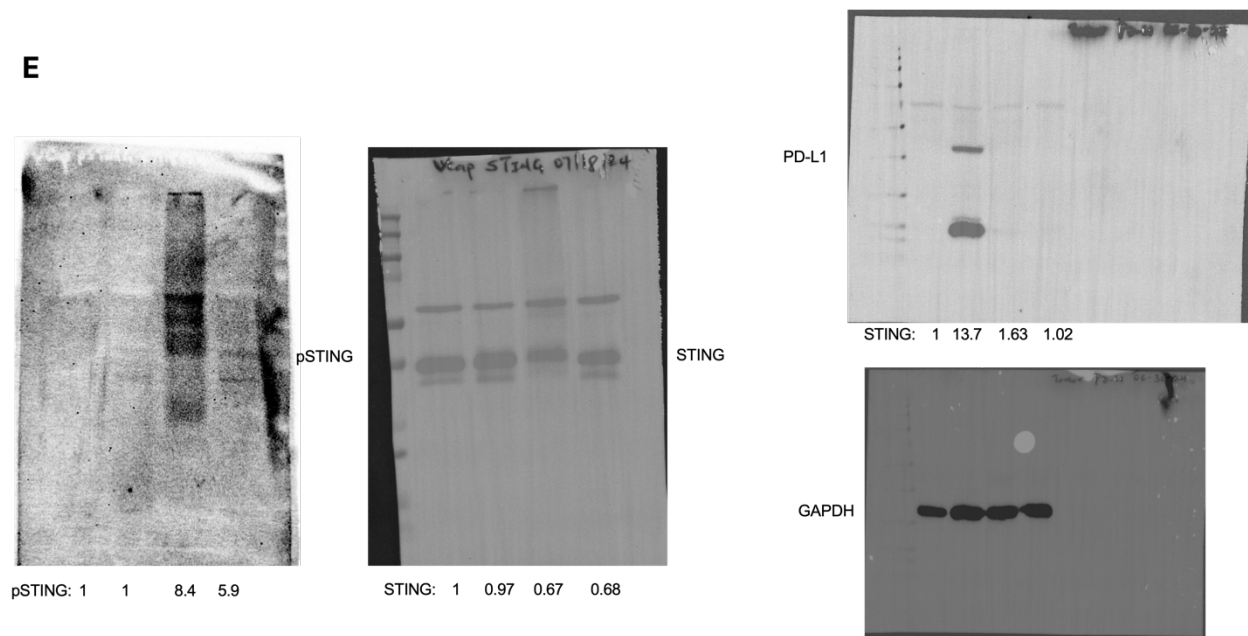

Supplement: Supplementary file 1 [file cancers-16-02918-s001.zip › PDF File. Original Western Blot images.pdf]
